# Cognitive and sensory capacity each contribute to the canine spatial bias

**DOI:** 10.1111/eth.13423

**Published:** 2024-02

**Authors:** Ivaylo Borislavov Iotchev, Zsófia Bognár, Soufiane Bel Rhali, Enikő Kubinyi

**Affiliations:** 1 Department of Ethology, ELTE Eötvös Loránd University, Budapest, 1117, Hungary; 2 Doctoral School of Biology, ELTE Eötvös Loránd University, Budapest, 1117, Hungary; 3 MTA-ELTE Lendület “Momentum” Companion Animal Research Group, Budapest, 1117, Hungary; 4 ELTE NAP Canine Brain Research Group, Budapest, 1117, Hungary

**Keywords:** cognition, dogs, spatial bias

## Abstract

Dogs interpret cues as being about location, which human infants would relate to objects. This spatial bias could shed light on the evolution of object-centered thought, however, research needs to rule out that this is not a by-product of dogs’ weaker (compared to humans) visual capacities. In this study, we used a data set in which dogs were tested in two types of learning tasks (discrimination and reversal learning) with two types of rewarded cues (location and object features). In both tasks, dogs displayed spatial bias, that is, faster learning when the rewarded cue was a location. We investigated how sensory and cognitive capacity each contributes to this spatial bias. To this end, an estimate for general cognitive ability (g) was obtained from a battery of tests for some of the dogs. Cephalic index, a feature targeted in breeding and linked to differences in visual capacity, correlated negatively with the expression of spatial bias only in the easier discrimination learning task, while a negative correlation between g factor and spatial bias scores emerged in the more difficult reversal learning task. We conclude that dogs’ spatial bias cannot be reduced to a sensory limitation and is easier to overcome with greater cognitive capacity.

## 1 Introduction

In comparing cognition across species, it is not only of interest which capacities are present or absent and to what degree but also which biases may guide information processing (see, e.g., discussed in Taylor et al., 2022). Dog cognition might be characterized by a “spatial bias” (also referred to in this way by Fugazza et al., 2016). Throughout different testing situations, dogs (*Canis familiaris*) appear to treat information as being about location rather than objects or object features. This was early on demonstrated in a delayed matching-to-sample task, wherein cueing a location, but not an object, was associated with above-chance performance (Dumas, 1998). Furthermore, while 9-month-old infants understand pointing as a reference to an object (Yoon et al., 2008), dogs seem to utilize the gesture as a directional cue (Tauzin et al., 2015). When imitating human actions, in the absence of additional instructions, dogs also more reliably copy the target location rather than the target object involved in the demonstration (Fugazza et al., 2016). Finally, dogs also learn more easily to associate a reward with a location rather than object features like the color or size of a plate (Piotti et al., 2018).

What could this apparent spatial bias teach us about the canine mind and, even more broadly, about the evolution of human-like cognition? To find out, it will be crucial to distinguish if it is truly cognitive or rather sensory in origin. In humans, for example, the ability to categorize objects has both perceptual and cognitive roots. Visual input plays a crucial role in the formation of proto-categories (Quinn & Eimas, 1997), conceptualizations that precede verbal categorization. Later on, language acquisition seems to further ease the capacity for categorization and object-related thought (Lupyan, 2006; Lupyan et al., 2007; Xu, 2002). To test the assumption that a spatial bias is inversely related to our capacity for object-related thought, we ought to investigate how it relates to sensory and cognitive capacities. A stronger relationship with cognitive capacity would support a role for spatial bias as an important marker of cognitive development (both onto- and phylogenetically). Although an allegedly cognitive spatial bias had been reported in nonhuman primates and 1-year-old children (Haun et al., 2006), the type of task used to test the subjects in that study could alternatively have been influenced by the A-not-B error, too (Piaget, 1971; Smith et al., 1999). Moreover, comparing primates might not be ideal for separating the sensory and cognitive factors at play, since many primate species are highly visual (discussed by Barton, 1998).

**Figure 1:**
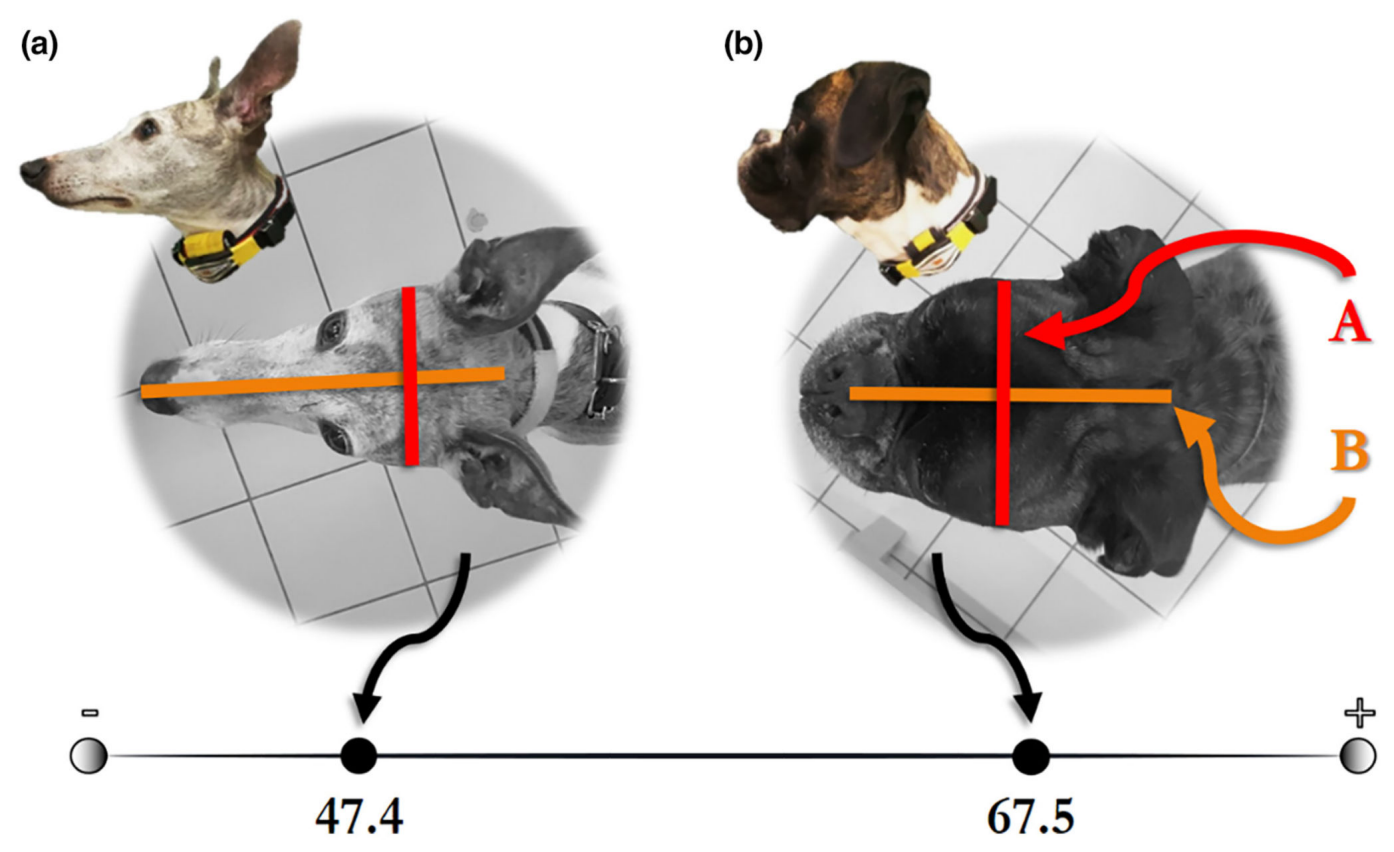
Examples of cephalic index (CI) values. CI is the ratio of the maximum width of the head (a) multiplied by 100 divided by the head’s maximum length (b). The shorter a dog’s head is, the higher the CI.

There is an intersection of reasons why studying the question in dogs is promising. First, they consistently express a spatial bias across many different tasks (Dumas, 1998; Fugazza et al., 2016; Piotti et al., 2018; Tauzin et al., 2015). Second, dogs are subject to a history of artificial selection which has resulted in divergent visual capacities across breeds, in connection with their head shape (Lind et al., 2017; McGreevy et al., 2004). The head shape of dogs can be quantified; its metric is the cephalic index (Evans & Lahunta, 2013). Cephalic index (further referred to as CI) is the ratio of the maximum width of the head multiplied by 100 divided by the head’s maximum length (see Figure 1). Shorter headed dogs (higher CI) are equipped with a higher density of retinal ganglion cells in the centre of their field of vision (resulting in higher visual acuity) and lower in the periphery, whereas longer headed dogs’ cells (lower CI) form a horizontally aligned visual streak of fairly even density (McGreevy et al., 2004). The latter arrangement suggests that longer headed dogs’ field of vision lacks a centered focus. The correlation between dogs’ CI and retinal ganglion cell density, combined with the emergence of binocular vision in short-headed dogs, allows this anatomic measure to function as a proxy for visual capacity. Further support for this operationalization comes from work confirming that differences in CI indeed account for differences in responding to visual stimuli (Bognár et al., 2018, 2021; Gácsi et al., 2009). Importantly, the literature distinguishes two approaches to quantifying CI differences—as a covariate (e.g., Bognár et al., 2021) or as categorical groups (e.g., Bognár et al., 2018). The latter has been criticized as arbitrary (Georgevsky et al., 2014), thus, here we quantify CI exclusively as a covariate.

A sensory hypothesis for the canine spatial bias can be derived from the assumption that visual constraints weaken attention toward stationary objects, increasing instead the attention to spatial relationships in the environment, which are easier to detect even with bad vision. In spite of a considerable inter-breed variability, dogs are by and large not on par with humans in the visual domain (see, e.g., differences in visual acuity directly compared by Lind et al., 2017). However, dogs with higher CI have a more human-like vision (sharper vision in the center of the visual field, binocular depth vision). We hypothesized that if spatial bias is purely sensory in origin, the variation in its expression should be sufficiently explained by variation in visual “hardware”, which varies naturally between dogs of different CI.

We can more directly inquire about the possible cognitive origin for dogs’ spatial bias by comparing its expression across dogs of different general cognitive abilities (referred to as g (factor) in humans, Spearman, 1904). Since a large subset of dogs in this study had participated in a wide range of cognitive tests (Table S1), it was possible to calculate g factor scores following rules outlined in the human literature (Bentler, 1990; Edwards & Bagozzi, 2000; Lorenzo-Seva & Ferrando, 2015). Although the external and construct validation of this canine g factor is an independently ongoing project (Bognár et al., 2023), this allowed us to create a summary variable for dogs’ overall cognitive capacity as expressed across a wide variety of tests. Notably, the individual subtests of the battery used for g factor extraction are supported in their validity by previously published works (Bognár et al., 2021; Kubinyi & Iotchev, 2020; Piotti et al., 2017).

While the literature allows for specific hypotheses about the role of sensory and cognitive factors in the expression of spatial bias, a more explorative approach was undertaken here with regard to demographic factors. Sex and age are consistently found to be associated with behavioral and physiological processes across a wide variety of domains, including visual attention, social responsiveness, sleep physiology, reactions to size-constancy violations, and marking behaviors (Beach, 1974; Bognár et al., 2018, 2021; Iotchev, Egerer, et al., 2019; Iotchev, Kis, et al., 2019; Kubinyi et al., 2009; Kubinyi & Iotchev, 2020; Müller et al., 2011).

A hypothesis regarding specific breeds emerges if spatial bias is deemed cognitive, reflecting a deficit in object-related cognition. We then predict a weaker spatial bias in breeds that show signs of operating more readily with object concepts. One impressive behavior in this regard is the ability attested in a small sample of dogs worldwide to use verbal and iconic object-referents in the absence of gestural guiding cues. Notably, Border Collies are currently overrepresented in this small circle (Fugazza, Andics, et al., 2021; Fugazza, Dror, et al., 2021; Kaminski et al., 2004, 2009; Pilley & Reid, 2011; Ramos & Ades, 2012), therefore, we decided to compare this breed with other homogenous breed cohorts (and accounting for head shape) on their affinity for spatial bias, although we simultaneously caution that the hypothesis rests upon very small numbers.

To pursue the question of whether the spatial bias is purely sensory, cognitive, or mixed in origin, we used a data set previously used to study discrimination and reversal learning, with two conditions in each (Piotti et al., 2018). In one condition, the animal was required to associate an object feature with a reward, whereas in the other condition, the cue was a location. We operationalize spatial bias as the ratio of trials needed to reach the criterion during the object feature condition compared with the location condition since more trials in the former are consistent with a greater difficulty in processing information as being about objects. Crucially, the reversal learning task appears to be harder for most dogs (Heckler et al., 2014; Mongillo et al., 2013), allowing us to further test if task difficulty plays a role in spatial bias. In Piotti et al., dogs also displayed spatial bias, but the phenomenon was not further examined directly. Here, in addition, 28 subjects were tested specifically for the current work and added to the existing data. We hypothesized that spatial bias is mixed in origin, with cognitive and sensory factors at play. We, therefore, expected that higher CI (associated with better visual capacity), a higher g factor score (better cognitive performance), and lower task difficulty would be associated with a weaker expression of spatial bias. In addition to these hypotheses, explorative motives prompted us to account for the possible involvement of sex, age, and breed in our analyses. Regarding the latter, a specific expectation about Border Collies expressing less spatial bias was borne out of their prevalence among the few worldwide examples of dogs learning object labels. Finally, we were also interested in whether the different conditions and associated difficulty could affect whether cognitive or sensory factors affect the expression of spatial bias more, expecting that cognitive capacity would be more crucial in the more difficult reversal learning condition.

## 2 Methods

### 2.1 Ethics statement

Our experiment is based on noninvasive procedures for assessing dogs’ behavior. According to the ethical statement issued by the Hungarian “Scientific and Ethical Committee of Animal Experiments” (PE/EA/2019-5/2017) and the corresponding legal definition, in Hungary, this noninvasive study is not considered an animal experiment. All owners gave written consent to participate with their dogs in the study.

### 2.2 Subjects

The subsample analyzed here consists of dogs from the study of Piotti et al. (2018) and dogs sampled specifically for this study (*N* = 28, valid *N* = 25), in total 82 dogs (39 ♀ [2 intact], 43 ♂ [12 intact], mean age ± SD: 9.5 ± 2.5 years, mean CI ± SD: 55.7 ± 8.3). Of these animals, 58 (27 ♀ [2 intact], 31 ♂ [10 intact], mean age ± SD: 9.2 ± 2.6 years, mean CI ± SD: 57 ± 9.1) continued with the reversal learning task after a preceding successful discrimination learning task. A prerequisite for participation was to meet the requirements of a sensory examination (Bognár et al., 2020). The largest homogenous breed cohorts in the data were Border Collies (*N* = 19), Vizslas (*N* = 17), and Whippets (*N* = 6). See our open-source data for further details on the breed composition of the sample.

### 2.3 STRANGE framework

Addressing the concerns outlined by Webster and Rutz (2020) in the STRANGE framework, we disclose that the sample used here consists exclusively of dogs kept as companions and from the territory of Hungary. It is, therefore, possible that some of the results will not generalize to, for example, stray dog populations or dogs kept in different cultures. Sampling bias can be excluded for the factors of age, sex, and breed.

### 2.4 Behavioral paradigm

**Figure 2:**
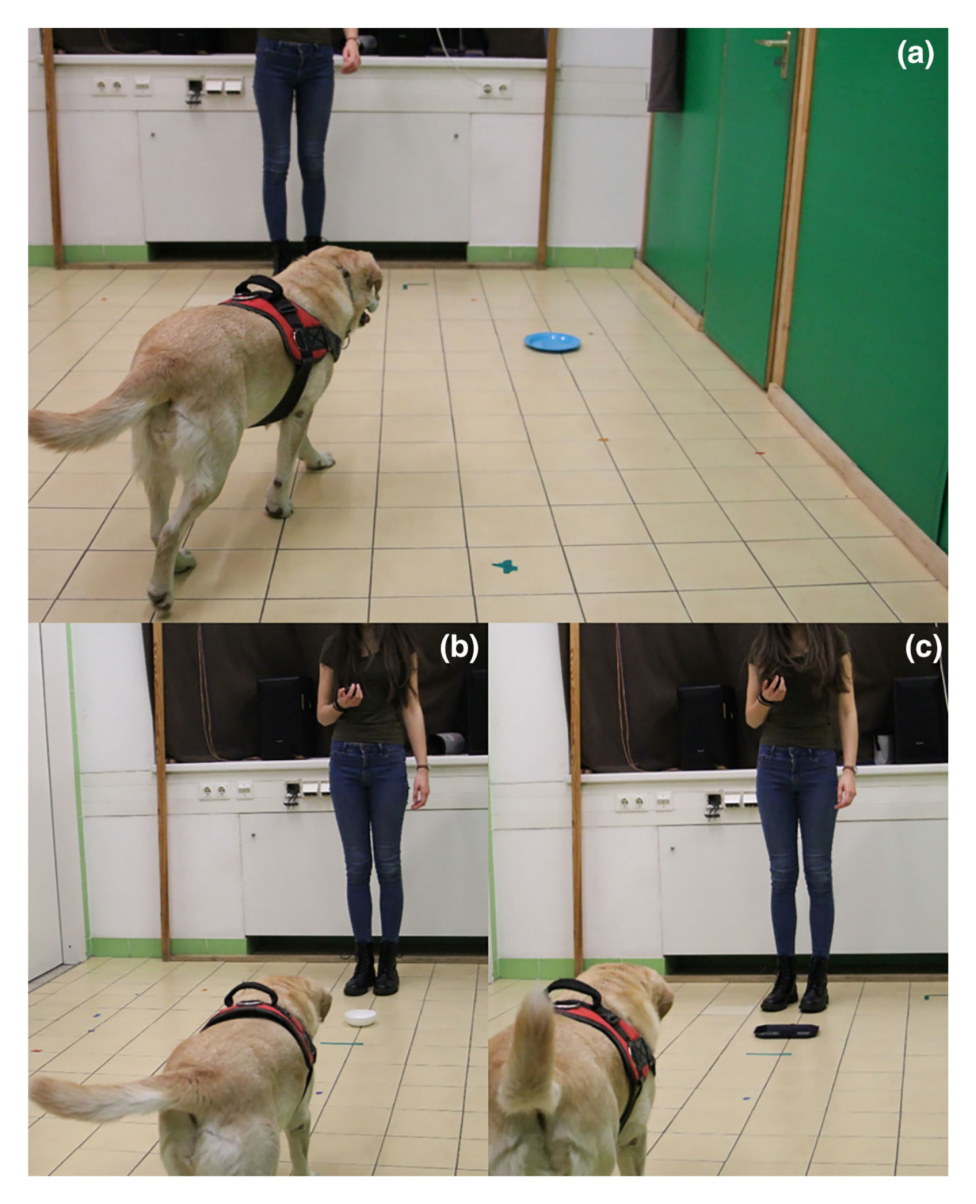
Spatial location condition (a) and object feature condition (b, c).

The testing environment was a small room (2.8 × 5 m) provided at the Ethology Department of the Eötvös Loránd University (Budapest, Hungary). During testing, only the animal, its owner, and an experimenter were present. The experimenter positioned themselves 3 m away from the dog-owner dyad, and apart from a chair for the owner, the room was empty. The animals were first trained to associate the spatial location or object features with the presence or absence of food (Figure 2). Specifically, in the spatial condition, the stimulus was the position of a round, blue plate (diameter: 20 cm) relative to the experimenter (left or right). The correct direction was left for half of the subjects. In the object feature condition, a plate was positioned in front of the experimenter, and its features were used as the predictive stimulus. Specifically, the presence of food was predicted either by a black, rectangular plate (23 × 15.5 cm) for approximately half of the subjects or a smaller, round, white plate (diameter: 12 cm) for the other half. All plates were made from plastic. The assignment to either learning condition was counterbalanced between dogs, and the conditions were switched between test and retest to avoid carry-over effects. Although previous results suggest that dogs do not rely on smell in similar set-ups (Szetei et al., 2003), all plates were smeared with food prior to testing.

In the spatial location condition, the task relied on egocentric spatial coding (i.e., the animal could rely on the representation of the objects in space relative to its own body axes, as along a left–right plane). Performance in this condition relied on spatial learning (discrimination task and reversal learning task) and executive function (reversal learning task). For the object feature condition, the performance relied on visual learning (discrimination task and reversal learning task) and executive function (reversal learning task). Both tasks also relied on visual discrimination-learning and reward and object approach-learning (domain).

Learning was measured in each task and condition through a series of consecutive trials, wherein the same stimulus type was allowed to repeat a maximum of twice in a row, following a pseudo-random order. In both cue conditions, only one stimulus type was used at a time. For each trial, regardless of condition or task, the dogs had 15 s time to reach the correct plate from the start of the trial and a maximum of 50 trials to reach the learning criterion within each condition and learning task. The owners were not allowed to make eye contact with the animals but could use short verbal encouragements if the dogs did not immediately start for the plate. A trial started when the experimenter placed a plate (baited or not) on the floor upon which the owner was instructed to release the dog. Learning criterion was reached when the longest latency for the correct choice within the last five trials was shorter than that of the corresponding previous latencies for the incorrect trials. After each trial, the measured latency was added to a spreadsheet which was programmed to notify if the learning criterion was reached by comparing the last five baited trials with the last five non-baited trials. For details of the paradigm not covered here, we also reference Piotti et al. (2018).

### 2.5 Statistical analyses and variables

A score for spatial bias was derived by calculating the ratio of trials to criterion between cueing conditions (object feature divided by location). This variable was a novel addition to the data set of Piotti et al. (2018). Likewise, a g factor score had been calculated for these subjects in a parallel investigation (Bognár et al., Preprint, 2023), however, not for the newly added subjects since they did not participate in the test battery (overview in Table S1) from which g was derived. These additional tests were omitted due to shortcomings associated with the COVID-19 crisis.

A generalized linear model (GLM) was used to test within each task condition (discrimination and reversal learning) and for each sequence of testing (object feature vs. location cue first) how spatial bias scores were associated with CI, g factor score, age, and sex of the animals. If assumptions of normality were violated for residuals (Shapiro–Wilk test of normality), a Gamma distribution assumption was specified, recommended for variables with all positive values. Next, for testing condition and cue effects, as well as breed cohorts (Border Collie, Hungarian Vizsla, Whippet), Wilcoxon-signed rank tests were used for paired comparisons, and Mann–Whitney *U* tests for independent samples. Nonparametric tests were chosen for these additional tests because the smallest sample in these comparisons was as low as *N* = 6 (Whippets in the breed cohort tests). From the relationship between sample size and the central limit theorem (Islam, 2018) follows that smaller samples are more likely to violate normality assumptions. All analyses were performed in SPSS v25.

## 3 Results

### 3.1 Condition effects

Spatial bias was observed in either task (discrimination and reversal learning), that is, dogs needed more trials to criterion with the object feature cue than the location cue in the discrimination learning condition (*Z* = −3.715, *p* < .001) and the reversal learning condition (*Z* = −4.847, *p* < .001). Spatial bias scores did not differ between learning task conditions, but a trend was observed for higher spatial bias scores in the reversal learning condition (*Z* = −1.715, *p* = .086).

Dogs who started training and testing with the object feature condition displayed significantly higher spatial bias scores in the discrimination learning condition (*Z* = −2.884, *p* = .004). However, no difference in spatial bias scores was observed between different starting cues in the reversal learning condition (*Z* = −0.866, *p* = .386).

### 3.2 Discrimination learning condition

**Figure 3:**
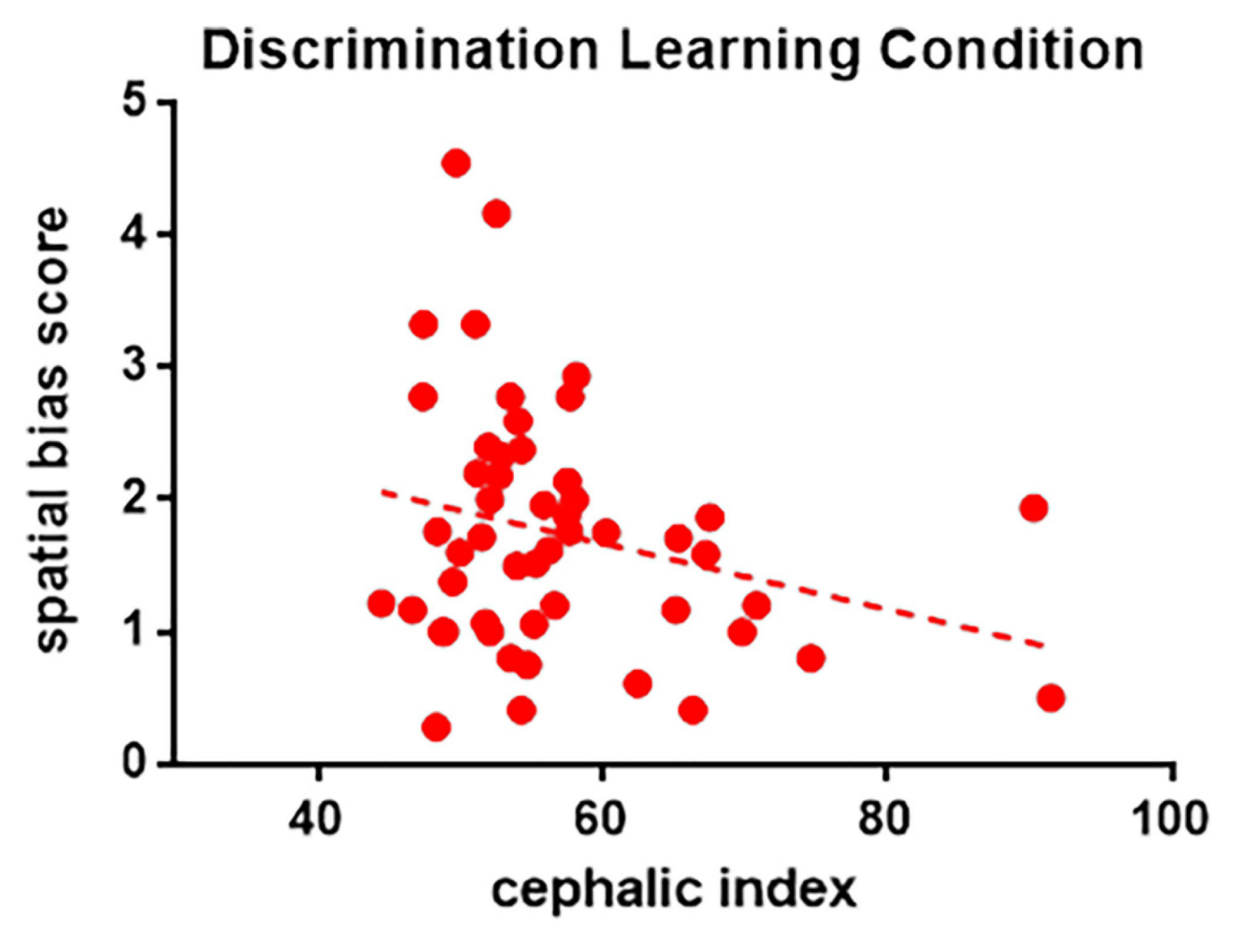
Spatial bias score as a function of cephalic index (CI), for dogs who started the discrimination learning task with an object feature cue.

For dogs who started with the object feature cue, CI was negatively correlated with the spatial bias score (GLM, Wald *χ*^2^ = 11.816, B = −0.038, *p* = .001, see Figure 3), but no associations were found for age, sex, or g factor score (*p* > .4). No associations with spatial bias score were found when the starting cue was the location (*p* > .05).

### 3.3 Reversal learning condition

**Figure 4:**
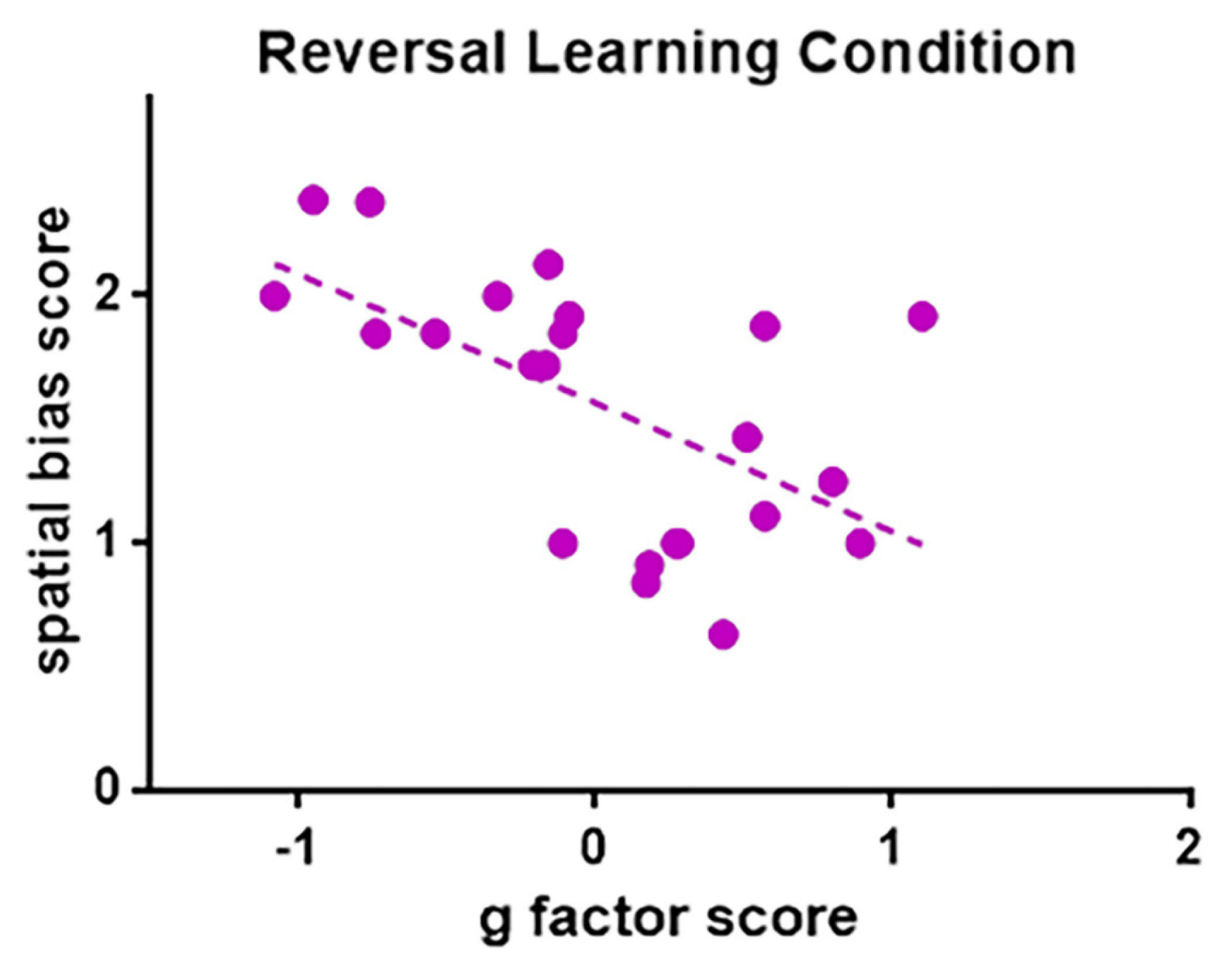
Spatial bias score as a function of the g factor score, for dogs who started the reversal learning task with an object feature cue.

For dogs who started with the object feature cue, g factor scores were negatively correlated with spatial bias scores (GLM, Wald *X*^2^ = 12.426, B = −0.541, *p* < .001, see Figure 4), but no associations were found for age, sex, or CI (*p* > .4). No associations with spatial bias scores were found for dogs who started with the location cue (*p* > .5).

### 3.4 Breed cohorts

The presence of spatial bias within breed-homogenous subsamples was tested by comparing trials to criterion for the object feature vs. the location cue. Only Whippets, Vizslas, and Border Collies were sufficiently big cohorts (*N* ≥ 6, see recommendation for minimum sample size by Camerlink & Pongrácz, 2021). To control for the above-described influence of head shape on spatial bias scores, we also compared CI values between these three cohorts (see Results in Appendix S1).

In the discrimination learning condition, Whippets displayed spatial bias (*Z* = −2.023, *p* = .043), but no difference between cues was found for Vizslas (*Z* = −1.119, *p* = .263) nor for Border Collies (*Z* = −0.039, *p* = .969).

In the reversal learning condition, spatial bias was displayed by Vizslas (*Z* = −2.383, *p* = .017) and Border Collies (*Z* = −2.493, *p* = .013), while too few Whippets were available for analysis in this condition (*N* = 4).

## 4 Discussion

In dogs, a spatial bias has been documented across many studies (Dumas, 1998; Fugazza et al., 2016; Piotti et al., 2018; Tauzin et al., 2015), but to our knowledge, never been studied directly before. In the present investigation, we addressed the question of how the phenomenon may be shaped by sensory and cognitive capacities, as well as the effects of artificial breeding. To this end, we used a data set in which a spatial bias had been previously reported (Piotti et al., 2018), but only in the reversal learning task, which was more difficult than the preceding discrimination learning task. In the present work, which included the addition of more short-headed dogs, Border Collies and Hungarian Vizslas, a significant spatial bias effect (faster learning when the relevant cue is a location) was observed in both tasks. This carries implications for possible follow-up work, suggesting that using easier tasks to study the phenomenon may require larger samples.

The results of our investigation strongly suggest that dogs’ spatial bias cannot be reduced to a sensory problem because, despite strong physiological (McGreevy et al., 2004) and behavioral indicators (Bognár et al., 2018, 2021; Gácsi et al., 2009) of better vision and increased visual attention in brachycephalic dogs, CI was neither the sole nor a persistent (across conditions) predictor of spatial bias scores. Here, our broadest observation about the phenomenon of spatial bias is that variables associated with its expression are more easily detected in subjects for which the task is novel when object-related cues need to be processed (i.e., for dogs who started the experiment with an object feature cue). The cue-type sequence had a significant effect on spatial bias scores in the discrimination learning condition, with higher spatial bias scores observed in dogs that started training and testing with an object feature cue. Moreover, associations between spatial bias scores with CI and g factor scores were also significant only for dogs whose first training cue was an object feature. This, by itself, betrays a more cognitive origin for spatial bias since prior experience with the task seems to diminish the effect of the cue type (object feature vs. location). That spatial bias scores were highest in dogs starting with the object feature cue may thus reflect how the absence of prior experience with the task amplifies the difficulty of the intrinsically harder learning from object features.

Specifically, CI was associated here (negatively) with spatial bias scores only in the easier, discrimination learning task. CI has been empirically linked to visual competencies like high acuity, depth vision, and visual attention (Bognár et al., 2018, 2021; Gácsi et al., 2009; McGreevy et al., 2004). Any known cognitive correlates of CI (Czeibert et al., 2020; Horschler et al., 2019) should also have been reflected in our g factor score for dogs (see Appendix S1), but since in our GLM analyses either only CI or g factor scores were significantly linked to spatial bias scores, we interpret CI here as a proxy for visual capacity. This implies that the contribution of sensory competencies to the expression of spatial bias is weak. It appears to provide brachycephalic dogs with a minor advantage against the bias, which, however, is lost when task difficulty increases (the reversal learning task was likely harder for the dogs based on work by Heckler et al., 2014; Mongillo et al., 2013; Piotti et al., 2018). Since brachycephalic dogs show signs of worse memory and self-control (Horschler et al., 2019), and generally high CI is associated with brain changes that may affect cognition adversely (Czeibert et al., 2020), it is possible that in the more difficult task, the advantage of good vision for high CI dogs was compensated by cognitive weaknesses. Therefore, although we only find direct support for the role of CI in the discrimination learning task, future attempts to study spatial bias in dogs need to account for the possibility that CI can be a source of variation, especially in easier tasks.

Overall, the cognitive nature of spatial bias in dogs is supported here by three observations. We already implied above the arguments stemming from the effects of cue sequence (object feature vs. location first) and task type (discrimination vs. reversal learning). These effects support the notion that spatial bias is influenced by experience with the task and task difficulty. Of these two, the role of task difficulty is supported only indirectly. Once by the distinct results obtained from analyses separately for each task condition, and by a trend that was observed for spatial bias scores being different across tasks. The third and most direct argument stems from comparing dogs’ spatial bias scores with a new estimate for general mental ability or g in this species (Bognár et al., 2023). G factor scores were available for a sub-sample of the dogs tested here (as not all subjects participated in the tests from which g was extracted). The observed effect suggests that a high g factor score is associated with lower spatial bias expression, an effect which became significant in the more difficult (Heckler et al., 2014; Mongillo et al., 2013) reversal learning task and for dogs who started training and testing with the object feature cue.

To what extent the canine spatial bias is the result of cognitive processes is a significant question, given the differences in visual sensory capabilities between human infants (who do not express spatial bias, Tauzin et al., 2015) and dogs (Lind et al., 2017). Although Haun et al. (2006) report different affinities for spatial bias within humans (infant development) and across primate species, which would suggest a sensory-independent origin for the phenomenon, these results could alternatively be explained by the A-not-B error. In other words, perseverance rather than a spatial cognitive bias might have explained the behaviors observed in their work. In this study, through our comparison with g factor scores, we present a more decisive case for spatial bias being a cognitive bias. A potential concern, that learning capacity rather than bias magnitude was reflected in the spatial bias score obtained for the reversal learning task, becomes possible if only object feature learning had become more difficult during this task. Our control analyses (Appendix S1) exclude this, demonstrating that during reversal learning, reaching criterion was significantly harder with each cue.

A more intriguing implication of the association between g factor and spatial bias scores is that within the species, dogs’ increased “intelligence” seems associated with a more humanlike (Tauzin et al., 2015) preference for the processing of object features. This interpretation of the results is exciting because of a controversy (discussed at length by Macphail, 1987) regarding whether we can compare intelligence across species in a rank-assigning manner. The fact that humans do not express spatial bias at all compared with dogs (Tauzin et al., 2015), but dogs of relatively lower intelligence (lower g factor) express it more, resonates with the layman’s notion of humans being “smarter” than most other animals. Likewise, the discovery that uniquely human levels of dendritic development in cortical pyramidal neurons correlate with IQ scores (Goriounova et al., 2018) suggests that some changes during human evolution enabled qualitatively higher cognitive processing compared to most other species. A more nuanced but not mutually exclusive argument is that some seemingly specialized modes of cognition, like object-centeredness, are emergent properties of a gradually increasing overall cognitive capacity. How this could work can be best understood by integrating the present results with those of Haun et al. (2006). In their experiment, 1-year-old human infants expressed a better memory for places than objects. The implication that humans may be born with a spatial bias, taken at face value (the previously mentioned limitations of their work noted), could mean that higher intelligence may help overcome this bias in the course of individual development.

In humans, there is substantial evidence demonstrating that object representations and object-centered thought may improve with the active acquisition and development of language (Lupyan, 2006; Lupyan et al., 2007; Xu, 2002). Another exciting area to explore, thus, emerges from the question of whether the role of language and intelligence in moving from location to object-centered cognition is mutually exclusive. Are these separate factors pulling development in the same direction, or could intelligence possibly play a role in the ontogenetic and phylogenetic emergence of language? Studies on individual dogs who excel at label-object associations may help answer this question in the future (Fugazza, Dror et al., 2021).

In addition to examining the contributions of sensory and cognitive competencies to the expression of spatial bias in dogs, we also set out to determine the possible effects of artificial breeding (partly addressed already by looking at CI), sex, and age. The demographic factors, age and sex, were eliminated during the optimization of our GLM analyses, and thus, the present work does not support a role for these factors in shaping the magnitude of spatial bias. We present some preliminary evidence for breed differences by comparing Whippets, Vizslas, and Border Collies, which differed from each other during the easier discrimination learning task. Only Whippets displayed spatial bias in this task, suggesting that some breeds may not be as susceptible to the bias as others. It is also interesting to note the absence of a spatial bias for Border Collies since they are over-represented among the small worldwide sample of dogs who readily learn label-object associations (Fugazza, Andics et al., 2021; Fugazza, Dror, et al., 2021; Kaminski et al., 2004; Pilley & Reid, 2011). However, several limitations currently preclude further speculation. First, this observation is based on very small samples, and while these are likely not underpowered (a spatial bias was detectable in the smallest sample, Whippets: *N* = 6), we could only exclude a possible interference with cue-type sequence in Border Collies and Vizslas (and only for discrimination learning, see Appendix S1 control analyses). We also cannot exclude that these differences reflect breed differences in CI (Appendix S1 control analyses). Finally, our results suggest that Vizslas could be similarly “immune” to this bias, thus, the possibility that Border Collies are a cognitively unique breed would require more evidence as to be seriously considered. Future efforts might unravel how spatial bias relates to the word-learning abilities of dogs in general and Border Collies in particular.

Overall, the present study offers crucial arguments and foundations for a deeper investigation into dogs’ spatial bias. First, our results reject the hypothesis of a purely sensory deficit and thereby confirm the relevance of follow-up efforts for the scholar of cognitive evolution. Second, we can derive several guidelines for the composition of future studies on this topic, which should avoid breed diversity (associated with head shape) and yet preferably aim for larger samples, especially when the tasks used involve easy, direct learning.

While we cannot exclude here the possible role of dogs’ sensory hardware during early development, the phenomenon seems to manifest on a more cognitive level of information processing in adult animals. One interesting question to pursue in the future is how the parallel processing of spatial and object-related information in the brain (Kolb & Whishaw, 2001) may underlie the emergence of a spatial bias.

## Supplementary Material

Appendix S1

Data S1

## Data Availability

The data that supports the findings of this study are available in the supplementary material of this article.
