# Supplementary material for: Cognitive and sensory capacity each contribute to the canine spatial bias
Source: Ethology. Author manuscript; Available in PMC 2024 Mar 1. (PMC7615695; doi:10.1111/eth.13423)
Supplement: Appendix S1 [file EMS194307-supplement-Appendix_S1.docx]

Cognitive and sensory capacity each contribute to the canine spatial bias

Ivaylo Borislavov Iotchev^a+1^, Zsófia Bognár^a1,2,3^, Soufiane Bel Rhali^1^, Enikő Kubinyi^1,3^

^a^ These authors contributed equally to this work

^+^ corresponding author: ivaylo.iotchev@gmail.com

^1^ Department of Ethology, ELTE Eötvös Loránd University, 1117 Budapest, Hungary

^2^ Doctoral School of Biology, Eötvös Loránd University, Budapest, Hungary

^3^ MTA-ELTE Lendület "Momentum" Companion Animal Research Group, Budapest, Hungary

**Supplementary**

*G factor – tests and extraction*

Cognitive tests from which the g factor was extracted are summarized in table S1 and belong to a cognitive battery developed for testing cognitive ageing [see Kubinyi and Iotchev^1^ for a shortened version of the battery]. The extraction followed standards from the human literature^2–4^ and is described in detail in Bognár et al.^5^ (Preprint stage). In a first step, PCA (principal component analysis) was run over the variables obtained for each subtest, using Oblimin rotation, as to create summary variables for the performances in each test. Polychoric correlation matrices were used if the variables measured for a given test were categorical. The summary variables were then also required to pass a test of retest-reliability, measured with ICC (two-way mixed model). Next, an exploratory factor analysis (EFA) was conducted across the obtained variables which indicated that eight tasks loaded on the first factor (putative g). The factor structure was further tested with confirmatory factor analysis (CFA) [see e.g. residual correlations criterion of Spearman^6^] which indicated a hierarchical structure with a single higher-order factor (g) at the apex. Construct and external validity of the g factor are the goal of an ongoing project (Turcsán et al. in prep). Here this summary variable was used in order to avoid cherry picking among the many potentially interesting cognitive tasks the dogs had participated in.

| **Name of Test** | **Cognitive Domain** | **Source/Example Literature** |
| --- | --- | --- |
| Manipulative persistency | persistency, motivation | ^7^ |
| Clicker game | associative learning, motivation | ^8^ |
| Problem solving | independence, inhibitory control | ^9^ |
| Attention | sustained attention | ^10,11^ |
| Training for eye contact | associative learning | ^8,10^ |
| Memory | Visuo-spatial short-term memory | ^12,13^ |

Table S1. Overview of the tests in the cognitive battery from which g was extracted. The EFA and subsequent CFA showed that Manipulative persistency, Clicker game, Problem solving, Training for eye contact, and Memory tests loaded strongly (> 0.3) with the g factor.

*Control analyses*

The three largest (N ≥ 6) breed cohorts were compared for differences in head shape (CI) to exclude that breed cohort differences in spatial bias scores are potentially explained by CI differences. CI was higher in the Border Collie cohort than in Vizslas (Z = -4.093, P < 0.001) and Whippets (Z = -3.467, P = 0.001). CI was also higher in Vizslas than in Whippets (Z = -2.38, P = 0.017).

We also were able to control for the effect of cue sequence on the expression of spatial bias in Border Collies and Vizslas, but not Whippets, since separately testing the presence of spatial bias for the different starting cues in Whippets would have created samples smaller than the recommended minimum of N = 6 ^14^. When the first cue was an object feature, in the discrimination learning task, no effect of cue on trials to criterion was observed in Border Collies (Z = -0.679, P = 0.497) or Vizslas (Z = -1.352, P = 0.176). For the reversal learning task and object feature starting cue, Border Collies displayed significantly more trials to criterion with the object feature cue (Z = -1.992, P = 0.046), while a trend for more trials to criterion with the object feature cue was observed for Vizslas (Z = -1.826, P = 0.068). When the first cue was a location, in the discrimination learning task, no effect of cue on trials to criterion was observed in Border Collies (Z = -0.674, P = 0.500) or Vizslas (Z = -0.420, P = 0.674). For the reversal learning task and location starting cue, no significant effect of cue was found in Border Collies (Z = -1.483, P = 0.138) or Vizslas (Z = -1.461, P = 0.144).

Another issue to control for was the possibility, that the correlation between g factor scores and spatial bias scores in the reversal learning task was primarily reflecting learning capacity rather than bias magnitude. This could be expected if only object feature learning had become harder in the reversal learning task, since this would make the relative difference in trials to criterion between the two cues meaningless. However, trials to criterion were significantly higher for each the location (Z = -5.027, P < 0.001) and object feature cue (Z = -6.527, P < 0.001) in the reversal learning task compared to the discrimination learning task.

**References**

1. Kubinyi, E. & Iotchev, I. B. A preliminary study toward a rapid assessment of age‐related behavioral differences in family dogs. *Animals* (2020). doi:10.3390/ani10071222

2. Edwards, J. R. & Bagozzi, R. P. On the nature and direction of relationships between constructs and measures. *Psychol. Methods* (2000). doi:10.1037/1082-989X.5.2.155

3. Lorenzo-Seva, U. & Ferrando, P. J. POLYMAT-C: a comprehensive SPSS program for computing the polychoric correlation matrix. *Behav. Res. Methods* (2015). doi:10.3758/s13428-014-0511-x

4. Bentler, P. M. Comparative fit indexes in structural models. *Psychol. Bull.* (1990). doi:10.1037/0033-2909.107.2.238

5. Bognár, Z. *et al.* The hierarchical structure of canine cognition: two domains and a general cognitive factor. *bioRxiv* (2023).

6. Spearman, C. ‘General Intelligence,’ Objectively Determined and Measured. *Am. J. Psychol.* (1904). doi:10.2307/1412107

7. Range, F. *et al.* The effect of ostensive cues on dogs’ performance in a manipulative social learning task. *Appl. Anim. Behav. Sci.* (2009). doi:10.1016/j.applanim.2009.05.012

8. Wallis, L. J. *et al.* Training for eye contact modulates gaze following in dogs. *Anim. Behav.* **106**, 27–35 (2015).

9. Gerencsér, L., Bunford, N., Moesta, A. & Miklósi, Á. Development and validation of the Canine Reward Responsiveness Scale -Examining individual differences in reward responsiveness of the domestic dog. *Sci. Rep.* (2018). doi:10.1038/s41598-018-22605-1

10. Chapagain, D. *et al.* Aging of attentiveness in border collies and other pet dog breeds: The protective benefits of lifelong training. *Front. Aging Neurosci.* **9**, (2017).

11. Wallis, L. J. *et al.* Lifespan development of attentiveness in domestic dogs: Drawing parallels with humans. *Front. Psychol.* **5**, 1–13 (2014).

12. Piotti, P. *et al.* The effect of age on visuo-spatial short-term memory in family dogs. *Pet Behav. Sci.* 17 (2017). doi:10.21071/pbs.v0i4.10130

13. Fujita, K., Morisaki, A., Takaoka, A., Maeda, T. & Hori, Y. Incidental memory in dogs (Canis familiaris): Adaptive behavioral solution at an unexpected memory test. *Anim. Cogn.* (2012). doi:10.1007/s10071-012-0529-3

14. Camerlink, I. & Pongrácz, P. Getting the statistics right for your manuscript. *Applied Animal Behaviour Science* (2021). doi:10.1016/j.applanim.2021.105333
